# Influence of surface defect density on the ultrafast hot carrier relaxation and transport in ${\hbox {Cu}}_2 {\hbox {O}}$ photoelectrodes

**DOI:** 10.1038/s41598-020-67589-z

**Published:** 2020-06-30

**Authors:** Lisa Grad, Zbynek Novotny, Matthias Hengsberger, Jürg Osterwalder

**Affiliations:** 1 Department of Physics, University of Zurich, Winterthurerstrasse 190, 8057 Zurich, Switzerland; 2 Paul Scherrer Institute, Forschungsstrasse 111, 5232 Villigen, Switzerland

**Keywords:** Electronic properties and materials, Surfaces, interfaces and thin films

## Abstract

Cuprous oxide (${\hbox {Cu}}_2 {\hbox {O}}$) is a promising material for photoelectrochemical energy conversion due to its small direct band gap, high absorbance, and its Earth-abundant constituents. High conversion efficiencies require transport of photoexcited charges to the interface without energy loss. We studied the electron dynamics in ${\hbox {Cu}}_2 {\hbox {O}}$(111) by time-resolved two-photon photoemission for different surface defect densities in order to elucidate the influence on charge carrier transport. On the pristine bulk terminated surface, the principal conduction bands could be resolved, and ultrafast, elastic transport of electrons to the surface was observed. On a reconstructed surface the carrier transport is strongly suppressed and defect states dominate the spectra. Evidence for surface oxygen vacancies acting as efficient carrier traps is provided, what is important for further engineering of ${\hbox {Cu}}_2 {\hbox {O}}$ based photoelectrodes.

## Introduction

The conversion of solar energy into fuel in photoelectrochemical cells (PEC) represents a sustainable way for energy conversion and storage, a typical route being the production of hydrogen via water splitting^1–3^. Upon light absorption in some semiconducting electrode material, photoexcited charges are generated that are separated and transported to the solid-electrolyte interface. Here they drive some catalyst-promoted redox chemistry that stores their energy in chemical bonds^4^. This concept promises to be both ecologically and economically attractive if electrodes with high conversion efficiencies can be built from cheap, Earth-abundant materials that are stable in the aggressive chemical environment of a PEC cell^5^.

Cuprous oxide (${\hbox {Cu}}_2 {\hbox {O}}$) has attracted much attention recently as electrode material for photo-electrochemical water splitting^6–10^. It is a p-type semiconductor with a band gap of 2.1 eV and has a maximum theoretical solar-to-hydrogen (STH) conversion efficiency of 18%^8^. The bare material is unstable towards reduction to metallic Cu under electrochemical hydrogen evolution conditions, but protective nanolayers of n-type ${\hbox {TiO}}_2$ or ZnO can stabilize the electrode. The alignment of the conduction bands and the band bending near the p–n junction make for the charge separation and transport of photoexcited electrons to the oxide-electrolyte interface^6^. ${\hbox {Ga}}_2 {\hbox {O}}_3$ nanolayers showed an even better band alignment for charge transport due to the very small conduction band offset. Recently a nanostructured photocathode design with ${\hbox {Cu}}_2 {\hbox {O}}/{\hbox {Ga}}_2 {\hbox {O}}_3/{\hbox {TiO}}_2$ buried junctions and with NiMo as hydrogen evolution catalyst was demonstrated^9^. It was implemented conformally in a coaxial nanowire structure to match the light absorption depth to the much shorter minority carrier diffusion length. It combines efficient light absorption with high positive onset voltage and shows high photocurrent density, paving the way towards efficient water splitting devices based entirely on Earth-abundant materials.

Electrode performance is very sensitive to the presence of interfacial or bulk defects, which typically form states within the semiconductor band gap. Photoexcited electrons may be efficiently trapped in these states. This dissipates free energy and reduces the electron mobility. A direct consequence is the reduction of the photovoltage that a semiconductor heterostructure can generate. Recently, Borgwardt et al. have used femtosecond time-resolved two-photon photoemission (tr-2PPE) in order to study the electron dynamics at the ${\hbox {Cu}}_2 {\hbox {O}}$-vacuum interface upon illumination with above-band-gap light^11^. On a reconstructed ${\hbox {Cu}}_2 {\hbox {O}}$(100) single-crystal surface, they observe ultrafast relaxation of photoexcited electrons from the conduction band into a copper vacancy bulk defect band with a decay constant of 110 fs. These charge carriers are transported towards the surface and populate a large density of long-lived surface acceptor states. These processes are proposed to substantially limit the obtainable photovoltage in ${\hbox {Cu}}_2 {\hbox {O}}$ devices^11^.

**Fig. 1:**
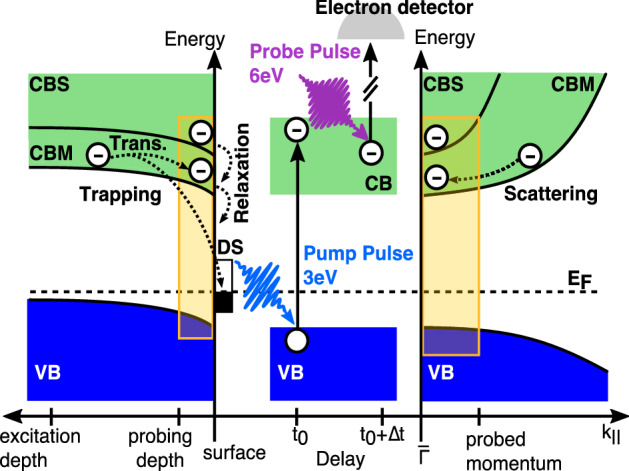
Principles of tr-2PPE. The bands and the electron dynamics at the surface are depicted in real space as function of depth (left), time delay in tr-2PPE (center), and in momentum space (right panel). By absorption of a pump photon, an electron is excited from the VB into the CB within the excitation depth. Subsequently, it can relax by scattering into lower-lying states and drift towards the surface, or else be trapped in a defect state DS. At the surface and at the band minimum close to ${\overline{\Gamma }}$ the electron can be emitted by absorption of a probe photon and eventually detected; the range accessible in 2PPE in real and momentum space is visualized by the yellow rectangles.

2PPE is a surface sensitive method in which a first laser pulse excites electrons from the valence band (VB) into the conduction band (CB), where the population is probed by a second laser pulse with well-defined, variable time delay on the femto- to picosecond time scale. The light absorption depth and the depth of the depletion region near the surface are much deeper than the probing depth of 2PPE. Therefore, the measured population dynamics reflects both, direct excitations within the surface region and transport of electrons to the surface in the electric field associated with the band bending. Furthermore, the momenta of photoelectrons which are probed by 2PPE lie in the vicinity of the minimum band gap at ${\overline{\Gamma }}$. Inelastic scattering of excited electrons will lead to an accumulation of electrons in these states close to the CB edge, which can be directly accessed by tr-2PPE (see Fig. 1). The lifetime of electron occupation in this band is one of the key parameters for the ultimately possible electrode performance.

Here we present time-resolved 2PPE data from a natural grown single-crystal ${\hbox {Cu}}_2 {\hbox {O}}$ surface with a different orientation, the (111) face. The surface was prepared in its unreconstructed, bulk-terminated ($1\times 1$) form with low surface defect density, as well as in a defect-rich reconstructed form with a ($\sqrt{3}\times \sqrt{3}$)$R30^\circ$ translational symmetry. The goal of this study was to distinguish between the effects of bulk and surface defects on the carrier dynamics. On the unreconstructed ($1\times 1$) surface we observe a delayed and strong population of the conduction band bottom that decays with a characteristic time of 10 ps. The reconstructed surface, on the other hand, shows no trace of carriers in the conduction band, but a filling of defect states over a period of about a picosecond. On the timescale of hundreds of picoseconds no noticeable decay was observed showing that these defects act as traps. These data suggest that the surface states, and in analogy also interface states, rather than the bulk defects limit the photovoltage in ${\hbox {Cu}}_2 {\hbox {O}}$ heterostructures. This is supported by recent observations of very high open-circuit voltages in ${\hbox {Cu}}_2 {\hbox {O}}/{\hbox {Ga}}_2 {\hbox {O}}_3$ heterojunction solar cells^12, 13^.

## Results

### Atomic and electronic structure of the (111) surface of ${\hbox {Cu}}_2 {\hbox {O}}$

**Fig. 2:**
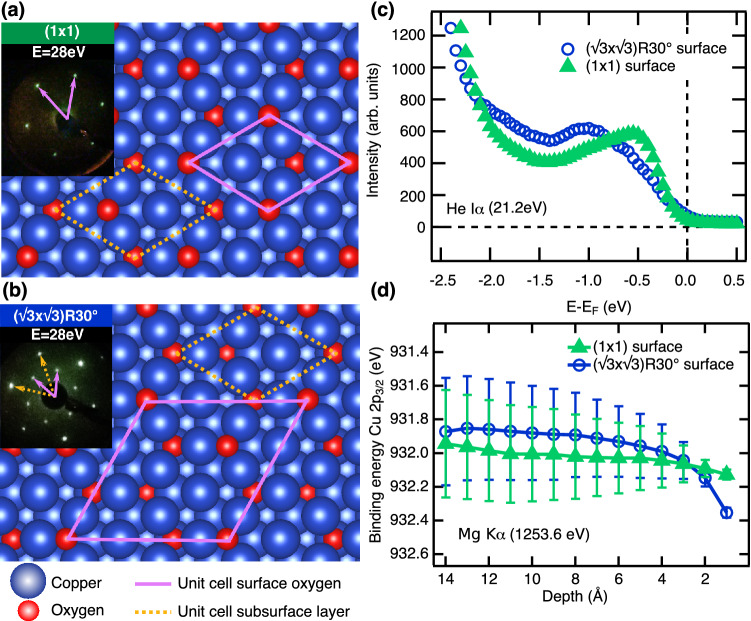
Atomic and electronic structure of the (111) surface of ${\hbox {Cu}}_2 {\hbox {O}}$. **(a)** Hardsphere model (copper: blue balls, oxygen: red balls) of the proposed surface structure of a bulk-like oxygen-terminated (1 $\times$ 1) surface and **(b)** of a surface containing ordered oxygen vacancies in a $(\sqrt{3}\times \sqrt{3}) {\hbox {R30}}^{\circ }$ reconstruction. The unit cells of the surface (pink lines) and of the subsurface layers (orange dashed lines) are indicated. Insets: LEED images taken at 28 eV from these surfaces. **(c)** The electronic structure close to the valence band maximum measured with He I$\alpha$ radiation of 21.2 eV, and **(d)** the band bending in the near-surface region as measured on the ${\hbox {Cu 2p}}_{3/2}$ core level using Mg K$\alpha$ radiation (1253.6 eV) for both surfaces: The (1 $\times$ 1) data are plotted as solid green triangles and the $\sqrt{3}$ data as open blue circles. The error bars in **(d)** were determined from the global fit.

Bulk ${\hbox {Cu}}_2 {\hbox {O}}$ crystallizes in the simple cubic structure with the Cu cations being in the oxidation state $+1$. It is unstable against further oxidation to Cu(II)O. As a result the oxide is naturally cation deficient, which results in p-doping of the bulk compound^14,15^. The atomic structure and stoichiometry of the ${\hbox {Cu}}_2 {\hbox {O}}$(111) surface depends on the preparation conditions, in particular on the annealing temperature. Two different, well-defined surface structures were investigated in the present work. We succeeded in switching the crystals reversibly between both surfaces by slight variations of the preparation parameters. In order to obtain the ($1\times 1$) surface preferably soft parameters for ${\hbox {Ar}}^{+}$ sputtering with an average ion kinetic energy of roughly 0.65 keV^16^ were chosen to reduce defect formation. Annealing at a temperature of $\approx 970~\hbox {K}$ leads then to the formation of a truncated bulk surface with an ($1\times 1$) translational symmetry. A hardsphere model assuming an oxygen-terminated surface and the corresponding low-energy electron diffraction (LEED) pattern at 28 eV are shown in Fig. 2a. Ion bombardment at higher ion kinetic energy (1.1 keV) followed by annealing at lower temperature (900 K) yields a different diffraction pattern corresponding to a $(\sqrt{3}\times \sqrt{3}) R30^\circ$ reconstruction, which will be referred to as $\sqrt{3}$ surface hereafter. The sharp LEED pattern shown in Fig. 2b already indicates that the additional defects introduced must be well ordered. Ion bombardement simulations using the SRIM package^17^ showed that under both preparation conditions the yield for oxygen removal is slightly higher than for copper and that an oxygen-deficient surface layer is created. During annealing the mobility of atoms and, thereby, vacancies is strongly enhanced while the mobility of sub-surface oxygen atoms is considerably lower than that for copper^18^. Diffusion leads then to a new crystalline equilibrium surface which depends on the exact preparation conditions (details see “Methods” section). For the $\sqrt{3}$ surface we propose that charged oxygen vacancies remain at the surface and form ordered structures due to mutual repulsion. This is in agreement with results of previous studies using photoemission and LEED^19^, and scanning probe microscopy^20^, in which oxygen vacancies were suggested to be at the origin of the $\sqrt{3}$ reconstruction.

For both surfaces the energy position of the valence band maximum (VBM) was measured using ultraviolet photoelectron spectroscopy (UPS) with He I$\alpha$ radiation (21.2 eV). The results are shown in Fig. 2c. For the bulk-terminated ($1\times 1$) surface the VBM is found at $E_\text{VBM}-E_{F}=(-0.10\pm 0.10)$ eV, where $E_F$ refers to the Fermi energy of the sample. The fact that the VBM is close to $E_F$ is in agreement with results of a combined X-ray photoemission and inverse photoemission study^21^ and can be rationalized by the intrinsic p-doping due to cation vacancies. In the case of the $\sqrt{3}$ surface a shift towards lower energies is observable, accompanied by the appearance of defect states in the gap just below the Fermi level, which makes the determination of the VBM onset difficult. Additional spectra taken with 6 eV laser light are included in the supplementary material of this work (Fig. S1). In this case the valence band maximum on the ($1\times 1$) surface is located at a lower energy of $E_\text{VBM}-E_{F}=(-0.27\pm 0.10)$ eV. Furthermore, the ionization energy can be determined as $E_\text{vac}-E_\text{VBM}=(5.62\pm 0.14)$ eV. In accordance to the He I$\alpha$ spectra, for the $\sqrt{3}$ surface the valence band maximum is shifted towards a lower energy of $E_\text{VBM}-E_{F}=(-0.41\pm 0.10)$ eV and the ionization energy is $E_\text{vac}-E_\text{VBM}=(5.54\pm 0.14)$ eV. Within the error bars the ionization energy is the same for both surfaces and is in agreement with previous studies^22^.

Due to the very short inelastic mean-free path for electrons at low kinetic energies, UPS probes only the band position close to the surface. The band bending in the near-surface region can be accessed using angle-resolved x-ray photoelectron spectroscopy (ARXPS). Here, the energy position ${\hbox {E}}_{0}$ of the Cu ${\hbox {2p}}_{3/2}$ core level is monitored as a function of emission angle $I(E, \theta )$ using a non-monochromatized Mg K$\alpha$ x-ray source (1253.6 eV). The angle-dependent spectra can be described as a sum over core level spectra $I(E, E_{0}(z), \Gamma )$ originating from different depths *z* with peak maxima located at $E_{0}(z)$ and an exponentially decaying intensity according to:

1 $$\begin{aligned} I(E,\theta )=\int _{0}^{\infty } \, I(E, E_{0}(z), \Gamma ) \, \exp \left[ -z/\Lambda _\text{in}(E_\text{kin}) \cdot \cos (\theta )\right] \, dz, \end{aligned}$$

where $\Lambda _\text{in}(E_\text{kin})$ is the inelastic mean free path at kinetic energy $E_\text{kin}$ of ${\hbox {Cu 2p}}_{3/2}$ photoelectrons^22^. The peak width $\Gamma$ is given by the intrinsic line width broadened by the experimental energy resolution, both of which are assumed to be constant. The function $E_{0}(z)$ obtained from a simultaneous fit of all angle-dependent spectra is directly related to the electrostatic potential $\phi (z)$ sensed by the Cu atoms in different atomic layers below the surface. The raw data (Fig. S2) and details to the fitting procedure are included in the Supplementary material.

**Fig. 3:**
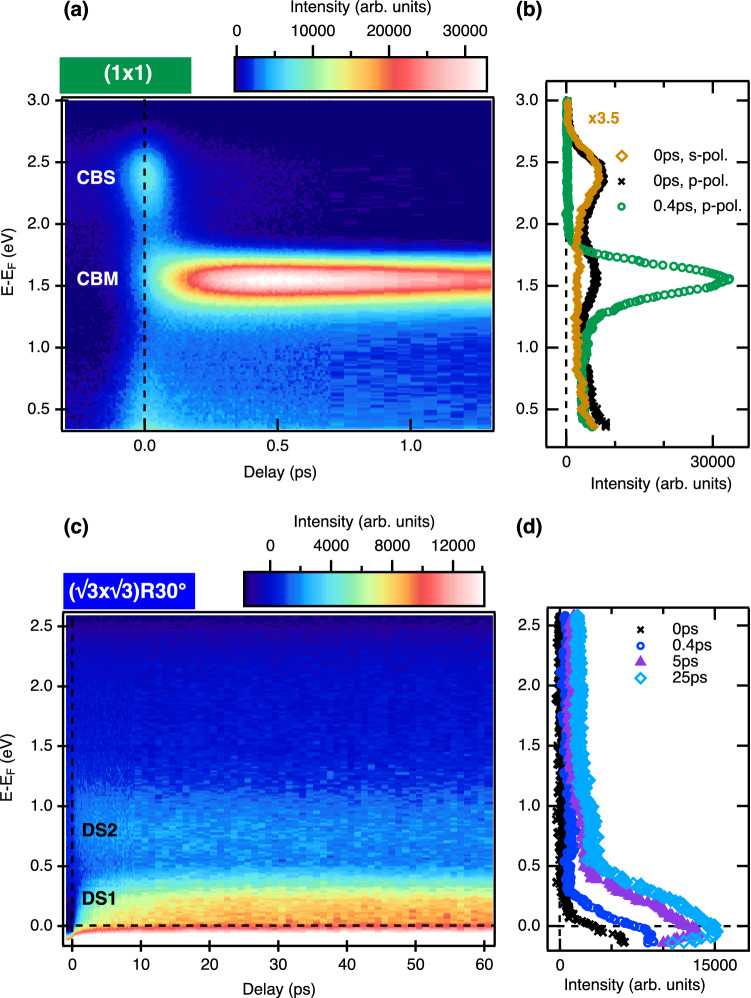
Transient electron-energy distributions. **(a)** Energy distributions after excitation with a p-polarized 3 eV pump pulse, color-coded as function of time delay (horizontal axis) and energy (vertical axis) for the ($1\,\times \,1$) surface with two conduction bands, CBS and CBM. **(b)** 2PPE spectra at coincidence (delay 0 ps) for s- and p-polarized excitation (solid brown diamonds and black crosses, respectively) and at 0.4 ps for p-polarized excitation (open green circles); the s-polarized data were scaled up by a factor of 3.5. **(c)** As in **(a)** but for the $\sqrt{3}$ surface. Two defect states, DS1 and DS2, are visible. **(d)** Spectra from **(c)** at selected delays.

In Fig. 2d the results for the two surface structures of ${\hbox {Cu}}_2 {\hbox {O}}$(111) are compared. The core level binding energy $E_{0}$ is plotted as function of the escape depth *z*. Due to the intrinsic p-type doping caused by bulk copper vacancies^23^ a downward band bending towards the surface can be observed in both cases. For the $\sqrt{3}$ surface with enhanced surface defect density an increased shift of around $\Delta E=0.45$ eV over the probing depth is noticed in comparison to $\Delta E=0.15$ eV for the ($1\times 1$) surface. This is in qualitative agreement with the observed shift of the VBM to lower energies in UPS shown in Fig. 2c, because the band bending causes a rigid shift of all energy levels^22^. The error bars are determined from the global fit. Due to the high surface sensitivity of this method, only a small part of the full width of the depletion layer can be investigated.

**Fig. 4:**
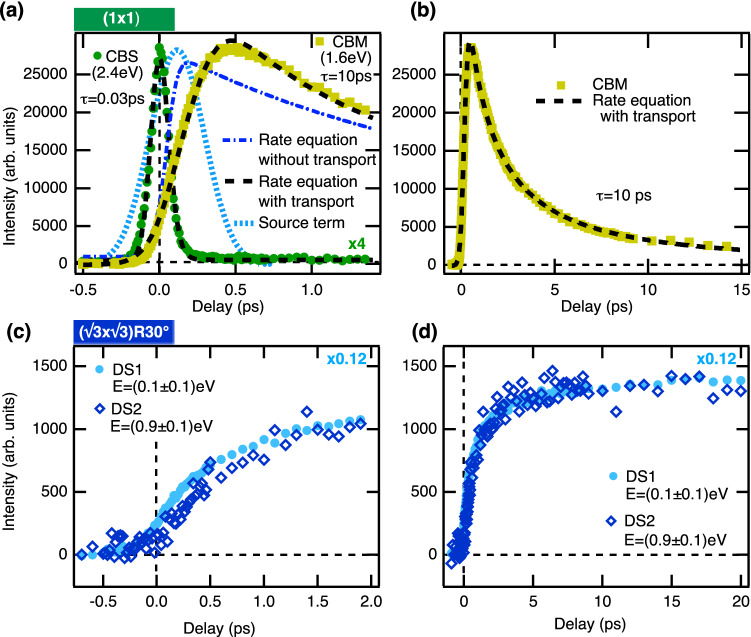
Population and relaxation dynamics of photoexcited electrons. **(a)** Integrated intensity of CBS (solid dark green circles), scaled up by a factor of 4, and of the CBM (solid light green squares) as function of time delay for the (1 $\times$ 1) surface. The data are fitted by rate equations (black dashed lines). For the CBM the result of the model without transport (dark blue, dash-dotted line) and the additional source term due to transport (light blue, dashed line) are shown for comparison. The time constants resulting from the fits are indicated. **(b)** Time-dependent CBM population on a longer time-scale. **(c)**, **(d)** Time-dependent integrated intensities of defect states DS1 (light blue solid circles) and DS2 (dark blue, open diamonds) from a $\sqrt{3}$ surface on short **(c)** and a long **(d)** timescales.

### Time-resolved 2PPE from a bulk-terminated surface

The electronic structure and the relaxation dynamics of photoexcited electrons between the Fermi- and the vacuum level around the ${\overline{\Gamma }}$-point were investigated by means of time-resolved 2PPE. For these measurements a 3 eV p- or s-polarized pump pulse with a fluence of $\Phi =33~\mu {\hbox {J/cm}}^{2}$ and a p-polarized 6 eV probe pulse were used at a pulse repetition rate of 100 kHz. The results for the bulk-like ($1\,\times \,1$) surface are shown in Fig. 3a,b. The color map shows the energy distribution of photoexcited electrons as a function of the time delay between both pulses. Two distinct features are observable. While the energetically highest state decays fast within femtoseconds, the lower lying state is filled within the same time scale and remains populated over picoseconds. To determine the energetic positions of these states, some selected spectra are plotted in Fig. 3b after subtraction of the background signal taken at negative time delays (for raw data see supplementary information, Fig. S3). The black curve shows the spectrum measured at zero delay for p-polarized pump light. Two peaks of similar intensities are visible with the maximum positions of $E-E_{F}=1.61$ eV and $E-E_{F}=2.37$ eV. These values can be referenced to the VBM position obtained from one-photon-photoemission (1PPE) using the same 6 eV laser probe light (Fig. S1) at $E-E_{F}=-0.27$ eV leading to $E-E_\text{VBM}=1.88$ eV and $E-E_\text{VBM}=2.64$ eV. It is noted in passing that the position of the VBM is unaffected by the presence of the 3 eV laser pulse. Comparing this to DFT calculations^24^ the states can be assigned to the conduction band minimum (CBM) and a higher conduction band state (CBS), respectively. While the energy position of the CBS coincides with the DFT calculation a larger deviation of the measured band minimum is observable. In Ref.^25^ values for the excitonic and electronic band gap of 1.9 eV and 2.1 eV, respectively, are reported. Therefore, the measured CBM feature probably corresponds to an exciton state. Following Ref.^26^ we locate the energy position of the CBM at the upper edge of the hydrogen-like series of states, which corresponds to the upper peak onset of the exciton signal. This yields a band gap of $E_\text{CBM}-E_\text{VBM}=2.12$ eV, in good agreement with published data^25^.

In comparison to the spectrum taken in temporal coincidence, the trace at 0.4 ps after the pump pulse looks dramatically different. The CBS peak completely vanishes on a very short timescale after time zero and cannot be recognized anymore in the spectrum taken at 0.4 ps. In contrast to that, the population in the CBM increases strongly and reaches its maximum roughly 0.4–0.5 ps after excitation. As can be seen from Fig. 3a and b, the CBM dominates the spectrum, and its intensity slowly decreases on a much longer picosecond timescale. In order to quantitatively analyze the relaxation dynamics of hot electrons in the conduction band, the transient intensities of CBS and CBM were extracted from the data and plotted as function of the time delay in Fig. 4. These so-called delay traces were fitted with the following kinetic rate equation model: For the CBS at $E-E_{F}= 2.37$ eV the time-dependent population $N_\text{CBS}(t)$ can be described as

2 $$\begin{aligned} \frac{dN_\text{CBS}}{dt} \,=\, A_{1} \cdot \exp \left\{ -\frac{(t-t_{0})^{2}}{2 \sigma _L^{2}}\right\} \, -\,\frac{N_\text{CBS}}{\tau _\text{CBS}} \end{aligned}$$

where $t_{0}$ denotes zero delay, $\tau _\text{CBS}$ the lifetime of photo-excited electrons in this state, $A_{1}$ gives the number of electrons excited by the pump pulse into the CBS, and $\sigma _L$ is the width of the pump pulse. The source term $A_1$ is proportional to the intensity of the pump pulse. The delay trace and the fitting result, which yields a lifetime of $\tau _\text{CBS}=0.030~$ps, are superimposed in Fig. 4a. A similar ultrafast relaxation within the conduction band towards the band minimum was already observed experimentally^27–29^ and theoretically^30^ for other metal oxides. It can be related to the emission of longitudinal optical (LO) phonons due to the large polarity of these materials, which results in a strong Fröhlich coupling. In cuprous oxide there are two possible LO phonon modes with energies of $E_{LO1}=19.1$ meV and $E_{LO2}=82.1$ meV^31^. The phonon emission rate at room temperature for these two modes can be calculated according to the model developed in Ref.^32^ by assuming an effective mass of $m^{\star }=0.985\, m_{e}$ and a high-frequency and a static dielectric constant of $\epsilon _{\infty }=6.54$ and $\epsilon _{S}=7.14$, respectively^31^. With this model we obtain relaxation times from the CBS into the CBM ($\Delta E\approx 0.8$ eV) due to phonon scattering of $\tau _{LO1}=0.067$ ps and $\tau _{LO2}=0.040$ ps, in good agreement with the observed relaxation time of 0.030 ps (for details see Supplementary material).

The interband scattering due to phonons has to be included as second electron source term in Eq. (2) when applied to the population of the lower lying CBM. This additional term must be proportional to the decay term of CBS, i.e. $\propto N_\text{CBS}/\tau _\text{CBS}$. Moreover, the decay of the CBM population cannot be described by a single exponential (Fig. 4). According to Ref.^33^ for excitons in ${\hbox {Cu}}_2 {\hbox {O}}$ an additional Auger-like two-body decay channel needs to be accounted for when using high excitation densities. Assuming an amplitude $A^{*}$ for the two-body decay we arrive at the following rate equation, which eventually was convoluted with the probe pulse:

3 $$\begin{aligned} \frac{dN_\text{CBM}}{dt}\,=\, A_{2}\cdot \exp \left\{ -\frac{(t-t_{0})^{2}}{2\, \sigma _L^{2}}\right\} \, + \,\frac{N_\text{CBS}}{\tau _\text{CBS}} \, -\, \frac{N_\text{CBM}}{\tau _\text{CBM}} \, - \, A^{*}\cdot N_\text{CBM}^{2}. \end{aligned}$$

Still, the fitted curve does not reproduce the experimental transient, because the instant of maximum population does not match the experimental value of $0.4-0.5$ ps as shown in Fig. 4a. So far, two channels to populate the conduction band minimum were neglected, (i) scattering in momentum space from states not visible in the measured region and (ii) drift of hot charge carriers excited in the bulk towards the surface (compare Fig. 1). The driving electric field is provided by the band bending within the depletion region like already assumed in a similar model in a recent study of ${\hbox {Cu}}_2 {\hbox {O}}$(100)^11^.

In order to exclude scattering in momentum space, measurements with s-polarized pump pulses were performed. For s-polarized light a direct transition from the valence band to the CBM is parity forbidden^23^. Moreover, the energetically higher CBS cannot be excited at large momenta using 3 eV pump light due to the dispersion of the valence and conduction bands^24^. In Fig. 3b the related spectrum is shown at the moment of excitation. As expected, only a direct excitation to the CBS is observable as for p-polarized light, but no excitation into the CBM. Yet, the time-dependent population of both conduction bands is qualitatively the same as in the p-polarized case, however with smaller yield (data are included in the supplementary material, Fig. S4). A slow filling of the CBM due to scattering with large momentum transfer can thus be ruled out leaving transport of hot carriers as the relevant mechanism.

The transport term can be obtained by numerically solving the equation of motion of an electron excited in a depth $z>0$ below the surface and subjected to the intrinsic electric field of the surface depletion region. The latter can be estimated from the angle-resolved XPS measurements which allow the potential gradient within the electron inelastic-mean-free path to be measured^22^. The results are shown in Fig. 2d for the two surfaces. The measurements can be fitted to the following generic potential function which results from the solution of Poisson’s equation at a semi-conductor junction^34^:

4 $$\begin{aligned} \phi (z>0)=\phi _{S}(0)\cdot \left( 1-\frac{z}{z_{0}}\right) ^{2}. \end{aligned}$$

Here $z_{0}$ is the width of the depletion region and $\phi _{S}(0)$ is the potential on the surface $z=0$. The numerical simulations are detailed in the Supplementary material. As a result, the transport can be approximated by a Gaussian with a broadened width $\sigma ^\prime$ and its peak position shifted by $\Delta t$ with respect to the gaussian-shaped source term of the laser pulse. The temporal population profile for the CBM can then be obtained using the rate equation Eq. (3) modified by an additional source term

5 $$\begin{aligned} A_{3} \cdot \exp \left\{ -\frac{(t-\Delta t)^{2}}{2\cdot {\sigma ^\prime }^{2}} \right\} . \end{aligned}$$

The result is superimposed to the experimental data in Fig. 4a and b where the transport source term is displayed by the blue dashed curve. In our simulations this source term corresponds to a width of the depletion layer of about 16 nm and a band bending surface potential of $\phi _{S}(0)=-0.1$ eV with respect to the bulk. These values agree well with values estimated from bulk properties for a bulk-terminated surface and with our band bending data shown in Fig. 2d, respectively (see Supplementary material for details). The decay constant obtained from the fit is $\tau _\text{CBM}=10$ ps. It should be noted that due to the two-body auger-decay the depopulation is distinctly faster for short delays after the maximum CBM population is reached ($\tau _\text{CBM}=2$ ps).

### Time-resolved 2PPE from a reconstructed surface

In a next step we investigate the role of surface defects on the electronic structure and the dynamics. The same measurements as before were performed on ${\hbox {Cu}}_2 {\hbox {O}}$(111) prepared with a $(\sqrt{3}\times \sqrt{3}) R30^\circ$ surface reconstruction. As outlined above, this reconstruction arises due to a dense array of well ordered surface defects. The transient spectra are shown in Fig. 3c. In stark contrast to the low-defect ($1\,\times \,1$) surface no photoemission signal from the conduction bands can be observed. Instead, an intense defect state (DS1) at the energy of about $E-E_{F}=0-0.1$ eV and a much weaker defect state (DS2) at an energy of $E-E_{F}=0.9$ eV dominate the spectra. The latter state DS2 was found on both types of surfaces and its intensity varies slightly between preparations (details in Supplementary information). Spectra taken at selected time delays are shown in Fig. 3d. Obviously, the peak of DS1 shifts towards higher energies with increasing time delay, suggesting the continuous filling of a surface-defect band. At the same time, the intensity of both defect states is steadily growing over picoseconds. The time evolution of the populations of the states DS1 and DS2 is shown and compared in Fig. 4c and d. After the slow rise, the intensity saturates at about 4 ps. No decay can be seen on these picosecond time scales.

## Discussion

The dramatic differences observed in the dynamics between the two surfaces confirm that carrier trapping in surface defects plays a key role in degrading the performance of cuprous oxide as electrode material. Here, the surface sensitivity of low-energy photoemission turns into an advantage as the signal intensity directly reflects the surface electron population. Our measurements clearly show that, in the case of a bulk-like surface, electrons excited deep inside the surface can drift within the conduction band to the surface where they create an energetic electron population that lives over tens of picoseconds. At energies of 2.12 eV above the valence band maximum, the electrons possess enough energy for driving the water splitting reaction if the surface can be rendered chemically stable by a suitable overlayer^9^.

In contrast, on the $\sqrt{3}$ surface containing a high density of ordered defects, no indication of electrons occupying the conduction band close to the surface was found. The spectrum is dominated by a long-living defect state (DS1) in the band gap. The second defect state (DS2), occasionally present on both surfaces, shows no influence on the drift and lifetime of electrons in the conduction band. Consequently, electrons excited into the conduction bands are efficiently trapped in low-energy defect states (DS1). The initial energy of the electrons is dissipated as heat, and the mobility of electrons is strongly reduced. As a result, any photochemical activity is suppressed.

The observed differences indicate that a high density of trapping defects can be introduced or removed within a few sputter and annealing cycles. Therefore, we conclude that the additional defects are localized in the near-surface region. Sputter yield simulations showed that an oxygen-deficient layer is created at the surface (details see “Methods” section). During annealing diffusion of atoms and defects increases strongly. Oxygen vacancies have by nature a much lower diffusion constant than copper vacancies^18^ and tend to remain close to the surface. In the topmost layer they show long-range order, which manifests itself in additional sharp LEED spots for the $\sqrt{3}$ surface. Recently, surface defects in ${\hbox {Cu}}_2 {\hbox {O}}$ were investigated by scanning tunneling microscopy and spectroscopy combined with DFT calculations^35^. The authors observed O-vacancies and predicted a mutual repulsion, as they carry a partial positive charge. Also the increase in downward band bending that we observe, which originates from additional positive charge in the topmost surface layer, is consistent with the presence of such oxygen vacancies. These observations confirm that, beside the intrinsic copper vacancies which are responsible for the p-doping of bulk ${\hbox {Cu}}_2 {\hbox {O}}$, the $\sqrt{3}$ surface additionally contains a high density of ordered oxygen defects which are responsible for trapping.

The role of oxygen vacancy states is contradictorily discussed in literature. While various first-principle calculations support our arguments and predict both Cu- and O-vacancy states in the vicinity of the VBM^36,37^ some photoluminescence (PL) studies assign a state close to the CBM to O-vacancies^38–41^ or to a vacancy complex^42,43^. There is a large variance of crystal growth conditions and consequently crystal defect properties. The appearance of this particular transition in PL depends on the growth conditions of synthesized crystals, films or nanoparticles, and was not observed on samples with low defect concentration^11,44^. Based on calculations, it was shown in Ref.^37^ that this PL signal can not stem from O-vacancies but could be related to a CuO surface layer, in agreement with results obtained on CuO/${\hbox {Cu}}_2 {\hbox {O}}$ samples^45^. This supports our assignment of DS1 to surface O-vacancies.

In a recent study a similar trapping mechanism was proposed to suppress transport in ${\hbox {Cu}}_2 {\hbox {O}}$ but invoking intrinsic copper vacancies^11^. According to calculations oxygen vacancies^36^ and so-called copper split vacancies^46^ generate localized hole states that are able to trap minority carriers. The appearance of particular defects and defect concentrations critically depend on the crystal properties and preparation conditions. High-temperature treatment under oxygen-poor conditions like in our case leads to loss of oxygen and eventually to a significant reduction in electric conductivity^14^. Therefore, oxygen vacancies are likely to be the dominating factor affecting the carrier mobility under reducing conditions. Moreover, in this work high-quality naturally-grown crystals were used, where the density of bulk defects is expected to be low^47^. We therefore conclude that the growth of ${\hbox {Cu}}_2 {\hbox {O}}$ electrodes with minimum amount of oxygen vacancies is a prerequisite for designing electrodes whose properties are limited by the properties of the interface only.

In photocatalysis transport of charge carriers photoexcited in the bulk towards the electrode-electrolyte interface without major energy loss is crucial to drive e.g. hydrogen evolution reactions. Therefore, long lifetimes and a high mobility of electrons close to the interface are important in order to enhance electron transfer rates, as for trapped charge carriers hopping between defect sites is the only transport mechanism possible. Moreover, our core level measurements pinpoint that the defect states created at the surface increase the band bending and thus reduce the built-in potential difference between electrodes in a photochemical cell. Cuprous oxide remains an excellent candidate material for solar-based applications. In the present study we could show that in surfaces with low defect concentration a persisting hot carrier population in the conduction band can be generated which lives long enough that the charges can reach the surface, or the interface to a protective layer. Under working conditions in a PEC cell such layers are required for chemical protection to preserve the performance of ${\hbox {Cu}}_2 {\hbox {O}}$ electrodes^7,11^. Our present findings on the effects of surface defects might allow a more specific design of those layers.

## Methods

### Sample preparation

Different naturally-grown and polished cuprous oxide (${\hbox {Cu}}_2 {\hbox {O}}$) single crystals (SurfaceNet GmbH) were used. The surfaces were oriented along the [111] direction. The data were recorded from different crystals, and all data sets were found to be consistent between the crystals and preparations. The surfaces were cleaned by a soft 30 min ${\hbox {Ar}}^{+}$ sputtering using a cold-cathode Penning source (ion current $I_{\text{Ar}}^{+}\approx 2~\mu$A as measured on the sample) and subsequent annealing under UHV conditions (30 min). The cleanliness and structural order of the surface were controlled by ultraviolet and x-ray photoelectron spectroscopy (UPS and XPS) and low-energy electron diffraction (LEED), respectively. After the first approximately 20 cleaning cycles all crystals showed the same and reproducible properties without any observable change over various preparations. Residual traces of carbon were found to be below 4% of a monolayer.

In order to switch reversibly between the ($1\times 1$) and the ($\sqrt{3}\times \sqrt{3}$)$R30^\circ$ surface structure different parameters for the preparation procedure were used. For the ($1\times 1$) surface sputtering was performed with the discharge source running without additional acceleration voltage. In this case, the average ion kinetic energy roughly corresponds to the discharge potential of $U=0.65~\hbox {kV}$^16^. The annealing temperature was $T=970~\hbox {K}$. We obtained the ($\sqrt{3}\times \sqrt{3}$)$R30^\circ$ surface by sputtering at higher energy and annealing at lower temperature. Specifically, we applied an additional acceleration voltage of $U\le 0.5 ~\hbox {kV}$ leading to an ion kinetic energy of about 1.15 kV, and the annealing temperature was reduced to $T=900~\hbox {K}$. During the switching procedure, an undefined transient surface structure was often observed, which disappeared after 5–10 cycles, when the new surface reconstruction appeared.

We carried out ion bombardment simulations in order to estimate the sputter yields for copper and oxygen using the SRIM package^17^. The simulations give removal yields of 0.9 and 1.26 copper atoms per ${\hbox {Ar}}^+$-ion and 1.16 and 1.62 oxygen atoms per ion at ion kinetic energies of 0.65 keV and 1.15 keV, respectively. This means that sputtering produces an oxygen-deficient surface layer in both cases. During annealing the mobility of the copper atoms is strongly enhanced and diffusion leads then to the new crystalline equilibrium surface. Note that the oxidation of Cu to ${\hbox {Cu}}_2 {\hbox {O}}$ proceeds via copper diffusion rather than oxygen diffusion, because typical mobilities for oxygen are smaller by several orders of magnitude than the mobility of copper atoms or likewise copper vacancies. The diffusion constant for Cu vacancies is $2.5 \cdot 10^{-6}~ {\hbox {cm}}^2$/s at 900 K which is to be compared to $4.2 \cdot 10^{-6}~ {\hbox {cm}}^2$/s at 970 K^18^. Due to the attractive interaction between oxygen vacancies and copper vacancies diffusion can also lead to compensation of these defects. The mobility increase with annealing temperature is significant and explains why the soft sputtering and high-temperature annealing results in bulk-terminated ($1\times 1$) surface structure. In contrast to that, for the preparation using sputtering at higher energy and annealing at lower temperature diffusion is reduced and an oxygen-deficient layer at the surface remains with oxygen vacancies as dominant defect in agreement with previous XPS studies^22^.

### Time-resolved photoemission experiments

For the time-resolved measurements a commercial mode-locked Ti:sapphire oscillator and a regenerative pulse amplifier (Coherent Mira Seed and RegA9050) were used. This system produces ultrashort laser pulses with a pulse duration of roughly $\tau =35$ fs at a tunable wavelength of $\lambda =780-830$ nm. The repetition rate was set to 100 kHz. The fundamental was frequency-doubled in a non-linear $\beta$-barium borate crystal to 416 nm (2.98 eV) and split into a pump and probe beam. While the pump beam was guided over a computer-controlled delay stage, the probe beam was again frequency-doubled in a second $\beta$-barium borate crystal to 208 nm (5.96 eV); the group velocity dispersion was compensated by a ${\hbox {CaF}}_2$ prism pair. The two beams were recombined using a dichroic mirror and focused co-linearly onto the sample. The $1/e^2$ beam waist of the pump pulse on the sample surface was around $500~\upmu \hbox {m}$ and the temporal width of the pulse intensity cross-correlation function was 90 fs full width at half maximum. The polarization for both beams could be adjusted using $\lambda /2$-waveplates between p- and s-polarization (electric field vector parallel or perpendicular to the plane of incidence on the sample, respectively). The optical reflectivity for 410 nm pump light is about $R^2 =17\%$ for p-polarized and $R^2 =38\%$ for s-polarized light at an incidence angle of $40-42^\circ$ from the surface normal^48^. Incident pump fluences between $\Phi =10-35~\upmu {\hbox {J/cm}}^{2}$ were applied, which correspond to maximum excitation densities of $5-18 \cdot 10^{18}~ {\hbox {cm}}^{-3}$ assuming an optical absorption length of 37 nm^48^. Due to an increased photoemission yield obtained from the $\sqrt{3}$ surface, the pump fluence for these measurements was reduced to typically $\Phi =10~\upmu {\hbox {J/cm}}^{2}$. Within the given fluence ranges, no charging or surface photovoltage effects were observed. The XPS data were taken in a modified VG ESCA Lab using the Mg K$\alpha$ line et 1253.6 eV^49^. In all other experiments, the photoelectrons were detected with a hemispherical electron analyzer (Specs Phoibos 100) equipped with a two-dimensional detector (intensity vs. energy and emission angle) and an acceptance angle of $\pm 15^{\circ }$^50^. For the measurements a bias voltage of $-10$ V was applied to the sample with respect to the analyzer. All measurements were performed at room temperature under ultrahigh vacuum conditions at a base pressure of $p\le 3 \cdot 10^{-10}$ mbar. The position of the Fermi level in the photoemission spectra was determined on a silver sample.

## Supplementary information

Supplementary information

## Data Availability

The datasets generated and analysed during the current study are available from the corresponding author on reasonable request.
