# Supplementary material for: Influence of surface defect density on the ultrafast hot carrier relaxation and transport in Cu2O photoelectrodes
Source: Sci Rep. 2020 Jun 30;10:10686. doi: 10.1038/s41598-020-67589-z (PMC7327060; doi:10.1038/s41598-020-67589-z)
Supplement: Supplementary file 1 — Supplementary information [file 41598_2020_67589_MOESM1_ESM.pdf]

Supplementary material for

Influence of surface defect density on the ultrafast hot carrier relaxation and transport in Cu_2O photoelectrodes

Lisa Grad[1], Zbynek Novotny[1,2], Matthias Hengsberger[1], Jürg Osterwalder[1*]

[1]University of Zurich, Department of Physics, Winterthurerstrasse 190, 8057 Zurich, Switzerland
[2]Paul Scherrer Institute, Forschungsstrasse 111, 5232 Villigen, Switzerland

[*]Correspondence to: osterwal@physik.uzh.ch

Supplementary Information:

- Supplementary Notes
- Supplementary Figures (S1-S7)

Valence band photoemission data

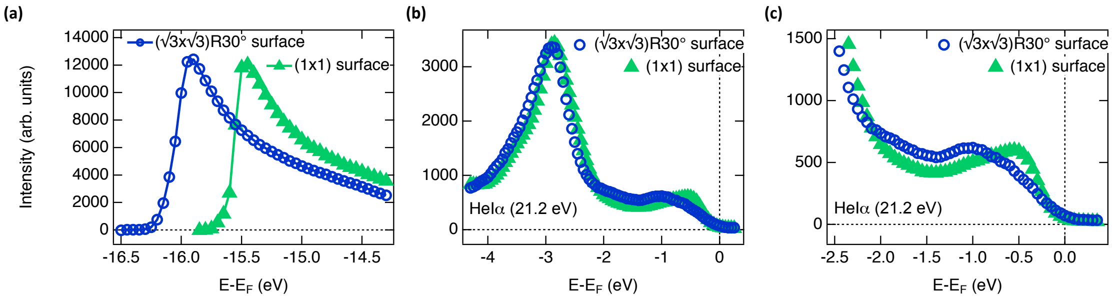

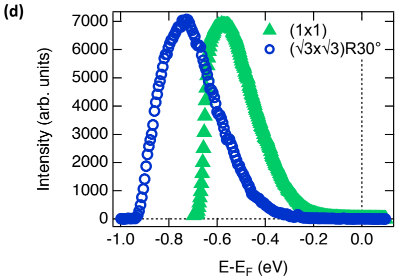

	1x1		(√3x√3)R30°	
E_{onset}-E_F (21.2eV)	-15.56eV	ϕ=5.64eV	-16.05eV	ϕ=5.15eV
E_{VBM}-E_F (21.2eV)	-0.10eV	E_{Ion}=5.74eV	-	
E_{onset}-E_F (6eV)	-0.65eV	ϕ=5.35eV	-0.87eV	ϕ=5.13eV
E_{VBM}-E_F (6eV)	-0.27eV	E_{Ion}=5.62eV	-0.41eV	E_{Ion}=5.54eV

Supplementary Figure S1: **Valence band spectra.** Compilation of valence band spectra recorded with different light sources for both surfaces: data taken from the (1×1) surface are plotted as green triangles, data from the $\sqrt{3}$ surface as open blue circles. (a) Low-energy secondary cut-offs (E_{onset}), (b) valence bands, and (c) bands close to the valence band maximum as measured with He Iα radiation of 21.2 eV. (d) Spectra taken with laser light of 6 eV. The values for the secondary cut-off E_{onset} and the upper valence band edge (VBM E_{VBM}), as well as the ionization energy E_{Ion} and the work function Φ are listed in the table.

Static one-photon photoemission (1PPE) spectra from the valence bands of $Cu_2O(111)$ measured with He Iα radiation of $h\nu = 21.2$ eV and laser radiation of $h\nu = 6$ eV from the two surface reconstructions are compared in Fig. S1. The position of the low-energy cut-off E_{onset} was determined by the energy position of the steepest rise (about the center) of the edge, the position of the valence band maximum (VBM) E_{VBM} by linear extrapolation of the falling edge. The Fermi level E_F was determined independently from a silver sample.

The work function can then by calculated as the difference of the photon energy and the width $E_F - E_{\text{onset}}$ of the spectra. The values are compiled in the table for the two surface reconstructions and the two light sources. The low-energy cut-off and thus the work function depends on the surface structure. For the work function, we obtain $\Phi = 5.35 \pm 0.10$ eV (6 eV) and $\Phi = 5.64 \pm 0.10$ eV (21.2 eV) for the (1×1) surface and a lower work function of

$\Phi = 5.15 \pm 0.10$ eV for the defect-rich $\sqrt{3}$ surface which is independent on the light source. This is in agreement with the hypothesis of surface oxygen vacancies being present in the latter case as the existence of defects with positive partial charge in the outmost layer induces a dipole opposite to the surface dipole, which has a net negative charge outside the surface [1]. This reduces the surface dipole and, thereby, the work function of the surface.

In Fig. **S1** b and c the valence bands are shown for both surface structures measured with He Iα radiation. While for the (1×1) case the onset of the VBM is just 0.1 eV below the Fermi-level a shift to lower energies can be recognized for the $\sqrt{3}$ surface concomitant with occupied defect states appearing below the Fermi level. These defect states make the determination of the VBM position unprecise. For spectra taken with 6 eV laser radiation (Fig. **S1** d) the tendency is qualitatively the same with a shift of the VBM to lower energies in case of a $\sqrt{3}$ surface. Nevertheless, due to the lower surface sensitivity at low kinetic energy, no signal of defect states below the Fermi level can be found in this case and the position of the VBM on the $\sqrt{3}$ surface can be determined as $E - E_F = -0.41$ eV. Furthermore, the absolute position of the VBM onset as measured with 6 eV is slightly different with $E - E_F = -0.27$ eV for the (1×1) surface. This shift was also observed in the low-energy cut-off meaning that it is a rigid shift of the complete spectrum. An explanation of this shift is still missing. It can not be explained in terms of band bending because for downward band bending a lower energy is expected for the more surface sensitive He Iα spectrum. Likewise a shift due to the surface photovoltage effect can be ruled out because the shift should occur in the opposite direction for p-doped material like Cu_2O.

Measurement of the near-surface band bending

Cu_2O exhibits intrinsically Cu vacancies, which act as electron acceptors. Thus Cu_2O is naturally p-doped and the Fermi energy is close to the VBM. At the surface of a semiconductor, the rearrangement of electronic charge and the presence of surface states and surface defects leads to a Fermi energy which is different from that of the bulk and thus to a diffusion of free majority carriers towards the bulk until the Fermi energy is equilibrated throughout the sample [2]. As a consequence of the transfer of free electrons in Cu_2O from the surface towards the bulk, the charge density gradient in the depletion layer produces a potential gradient which, in turn, leads to a downward band bending towards the surface. The width of the band bending region is related to the effective hole concentration and the dielectric screening by [2]:

$$\phi_S = \frac{e\, p\, z_0^2}{2\, \epsilon},$$

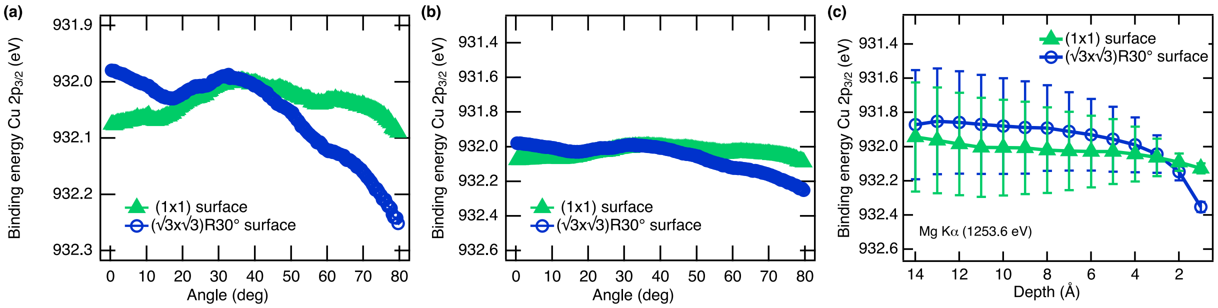

Supplementary Figure S2: **Band bending.** (a) The binding energy of a Cu $2p_{3/2}$ core level measured with Mg Kα radiation of 1253.6 eV is plotted as a function of the emission angle in detail and (b) on a larger energy scale. (c) After proceeding a global fit analysis based on an integration model the binding energy dependent on the probing depth can be obtained.

where ϕ_S is the surface potential, which equals the total band bending over the width z_0 of the depletion layer, p the effective free hole concentration, and ϵ the static dielectric constant. Using $p \approx 10^{12}$ cm^{-3} at room temperature and $\epsilon = 7$ [3] we obtain a width z_0 of the order of nanometers for a surface potential of the order of $\phi_S \approx -0.1$ eV.

Using angle-resolved x-ray photoelectron spectroscopy (ARXPS) the near-surface part of the band bending can be probed by measuring the binding energy of a localized core level E_0 as function of the emission angle θ [4] (see Fig. **S2** a,b). The core level line $I(E, \theta)$ recorded at a polar emission angle θ can be described as signal integrated over contributions originating from different depths z with exponentially decaying intensity:

$$I(E,\theta) = \int_0^\infty I\left[E, E_0(z), \Gamma\right] \exp\left\{-z/\left[\Lambda_{\text{in}}(E_{\text{kin}}) \cdot \cos(\theta)\right]\right\} dz, \tag{1}$$

where $\Lambda_{\text{in}}(E_{\text{kin}})$ denotes the inelastic mean-free path at kinetic energy E_{kin}. The product $\Lambda_{\text{in}}(E_{\text{kin}}) \cdot \cos(\theta) = \langle z(\theta) \rangle$ represents the effective mean escape depth $\langle z(\theta) \rangle$ of elastic electrons emitted from the sample with a polar emission angle θ.

In order to extract the band bending $E_0(z)$ from the measured angle-dependent spectra the whole set of ARXP spectra was fitted with Eqn. 1 using emission from discrete layers every Ångstrøm from the surface $z = 0$ down to a depth, which corresponds to twice the inelastic mean-free path $z_{\text{max}} = 2\,\Lambda_{\text{in}}$. The inelastic mean free path was estimated using the TPP-2M model [5] to be $\Lambda_{\text{in}} = 7$ Å for the Cu $2p_{3/2}$ core level at a kinetic energy of 320 eV. Assuming that each layer provides a gaussian-shaped contribution with exponentially decaying amplitude a sum of all these contributions is fitted to each spectrum $I(E, \theta)$. A Shirley-background was subtracted beforehand, and all 15 spectra taken at different emission angles $\theta = 0°, 5°, \ldots 70°$ were analyzed using a global fit procedure. The peak width Γ is given by the intrinsic line width broadened by the experimental energy resolution, both of which are assumed to be constant.

$$I(E,\theta) = \sum_{n=1}^{14} I\left[E, E_0(z_n), \Gamma\right] \, \exp\left\{-z_n / \left[\Lambda_{\text{in}} \, \cos(\theta)\right]\right\}.$$

From this global fit model we obtain the binding energy E_0 of the Cu $2p_{3/2}$ core level as function of depth z and the error of the obtained values could be determined (Fig. **S2** c). Since the energy of a localized core level depends essentially on the electrostatic potential, the energy dependence on escape depth can be translated into a depth-dependent potential [4], as shown in Fig. **S2**.

Data treatment for the presentation of time-dependent data

The time-resolved 2PPE data presented in this work (e.g. Fig. **S3**) are difference data obtained by subtracting the equilibrium energy distribution from all transient spectra. The equilibrium spectra were calculated from the raw data by averaging all spectra taken at negative delays, i.e. for the probe pulse preceding the pump pulse, between very long negative delays and delays short before the onset of the pump pulse. This average signal was subtracted from the measured data. For comparison the raw data and the corresponding difference data are shown together in Fig. **S3**. One can easily see that this correction does not affect the measured features. Moreover, as a positive side effect, the structureless and delay-independent background of thermally excited electrons, which can be seen in the three-dimensional representation and which obscures some small transient changes, is subtracted from the data.

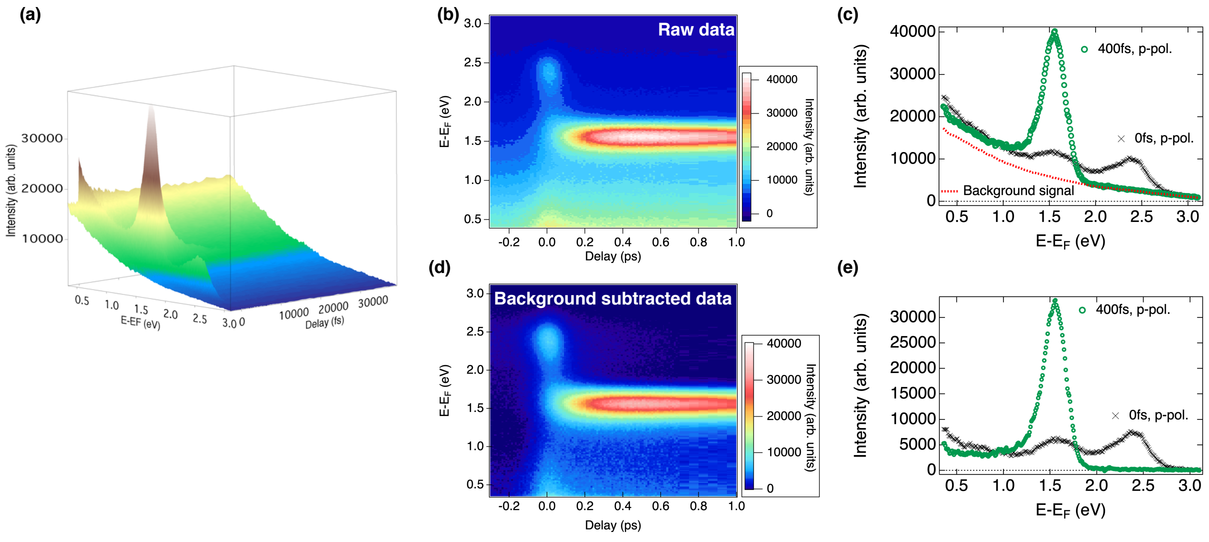

Supplementary Figure S3: **2PPE difference spectra.** (a) Raw data of a time-resolved 2PPE experiment, shown as three-dimensional dataset 2PPE intensity versus time delay and energy. (b) Same data set than in (a) but as false-color plot. (c) Two selected spectra out of the dataset in (a). The background due to thermally excited electrons is indicated by a red dashed line. It corresponds to the averaged spectra at negative delays. (d) As in (b) but after subtraction of spectra at negative delays. (e) As in (c) after removal of the background.

Electron-phonon coupling

In polar semiconductors like Cu_2O electrons interact strongly with the electric field caused by optical phonons leading to the formation of a polaron. This phenomenon was solved quantum-mechanically by H. Fröhlich [6]. The strength of this electron-phonon interaction is given by the dimensionless so-called Fröhlich coupling constant

$$\alpha = \frac{e^2}{\kappa\, \epsilon_0} \cdot \sqrt{\frac{m^\star}{2\hbar^3 \Omega}}$$

with the electron effective mass $m^\star$, the optical phonon frequency Ω and the dimensionless parameter $\kappa^{-1} = \epsilon_\infty^{-1} - \epsilon_S^{-1}$ which depends on the static and high-frequency dielectric constants ϵ_∞ and ϵ_S. One consequence of this coupling is an efficient energy transfer from the electrons to the lattice. The relaxation rate τ^{-1} of an excited electron into an energetically lower lying

state with the energy difference ΔE can be calculated according to:

$$\frac{1}{\tau} = \frac{e^2\,\Omega\,\sqrt{m^\star}}{4\pi\,\epsilon_0\,\hbar\,\sqrt{2E}} \cdot \frac{1}{\kappa} \cdot \ln\left(\frac{\sqrt{\Delta E} + \sqrt{\Delta E - \hbar\Omega}}{\sqrt{\Delta E} - \sqrt{\Delta E - \hbar\Omega}}\right) \cdot \left(1 + \frac{1}{\exp\left(\frac{\hbar\Omega}{k_B T}\right) - 1}\right)$$

Taking the material parameters for Cu_2O $\epsilon_\infty = 6.54$, $\epsilon_S = 7.14$, $m^\star = 0.985 \cdot m_e$ and the longitudinal optical phonon energies $\hbar\Omega_1 = 19.1$ meV, $\hbar\Omega_2 = 82.1$ meV [7], as well as the experimental energy difference $\Delta E = 0.8$ eV for a relaxation from the higher conduction band state (CBS) towards the conduction band minimum (CBM), we obtain relaxation times of $\tau_1 = 67$ fs and $\tau_2 = 40$ fs for the two phonon branches. These timescales match well the fast decay of CBS observed in the experimental data, which, together with the strong coupling strength explain the fast decay within 30 fs towards the conduction band minimum by an interaction with longitudinal optical phonons.

2PPE data with s-polarized light

In addition to the data presented in the main part where the excitation was done with p-polarized light, the same measurements were performed with s-polarized excitation pulses. In case of p-polarization the electric dipole operator acting on the initial state is symmetric with respect to the plane of incidence, while it is anti-symmetric for s-polarized light. If the plane of incidence is a plane of symmetry of the crystal, the wavefunctions have a well-defined parity with respect to this symmetry plane. Due to optical selection rules only transitions between states of different symmetries with respect to the plane of incidence are allowed for s-polarized light.

The valence band of Cu_2O consists predominantly of Cu 3d states and the conduction band of Cu 4s states, which both are symmetric under reflection at the mirror planes of the crystal lattice. Therefore, a direct transition between these states of same symmetry is forbidden for s-polarized excitation. This allows us to suppress contributions from direct transitions from the VBM into the CBM in 2PPE, and thus to suppress the population of the CBM at the $\overline{\Gamma}$-point due to intraband scattering. As a consequence, transport from electrons photo-excited in the bulk towards the surface due to downward band bending is the only effect which leads to a *time-delayed* increase within the probed surface region (see Fig. 1). In Fig. **S4** the polarization-dependent results are compared: The only difference, except for the smaller photoemission yield for s-polarized pump light, is the spectrum taken at coincidence of pump and probe pulse. Here only an excitation into the higher conduction band (CBS) is observed for s-polarized light, whereas the direct transition into the band minimum (CBM) is parity forbidden. For longer delays, the time-dependent results are qualitatively identical, i.e. the transient population of the

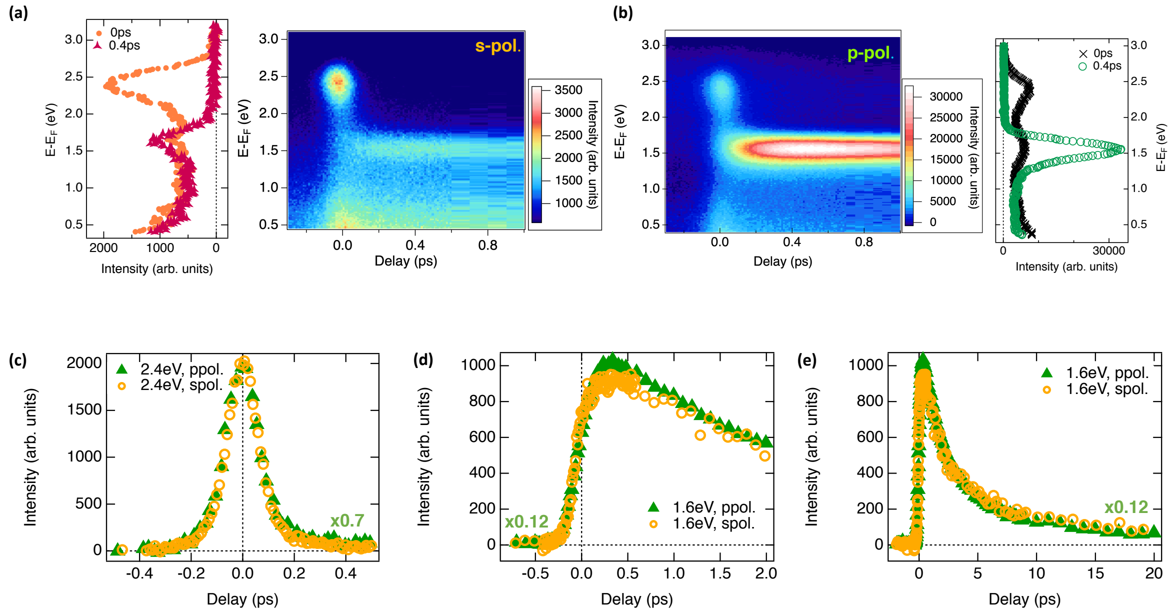

Supplementary Figure S4: **Data for s-polarized pump light.** The time-dependent energy distributions after excitation using (a) s-polarized or (b) p-polarized pump light with a photon energy of 3 eV are compared. Spectra at zero-delay and 0.4 ps after excitation are shown in the side panels. The transients of the two conduction bands for both light polarizations are superimposed in (c) for the state CBS, and (d) and (e) for the CBM on a short and long time scale, respectively. The intensities of the transients with p-polarized light were normalized to those of the s-polarized data.

CBM at $\overline{\Gamma}$ rises slowly and reaches its maximum about 400 fs after the pump pulse. From this we conclude that intraband scattering within the CBM plays a minor role and can be neglected for the quantitative analysis by the rate equation model.

Electron transport simulations

In general, 2PPE transients in non-interferometric setups can be modeled by using first order differential equations like Eqn. 3. The change in occupation number of the states is then solely determined by the rates of population and decay of these states. The latter leads to a simple exponential decay. The first term is called the source term and can have arbitrary form if the

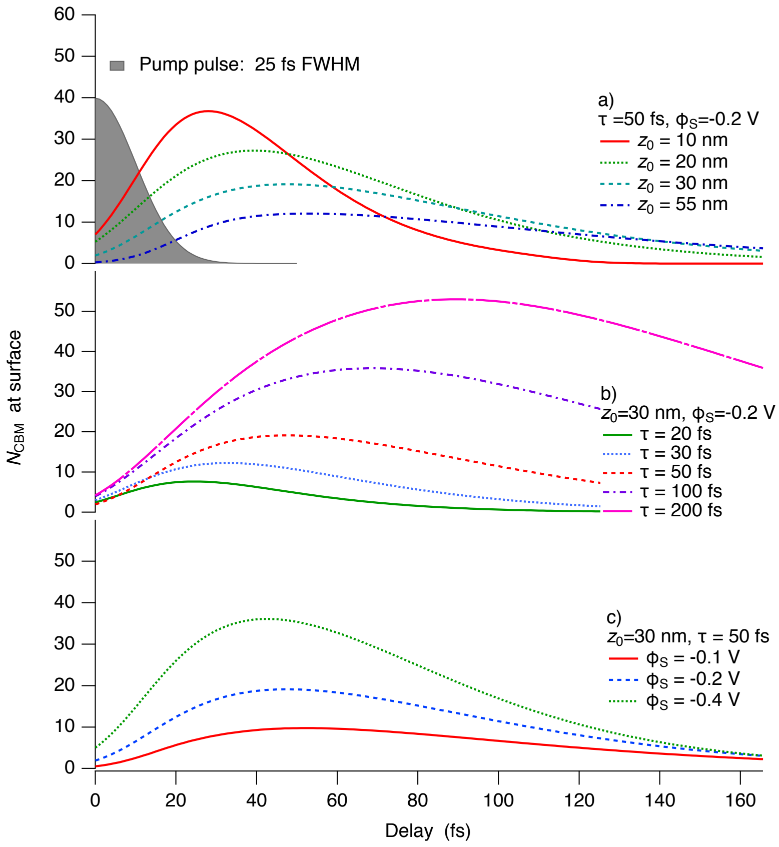

Supplementary Figure S5: **Simulation of the transient CBM population at the surface due to transport.** Electrons are excited over the width of the depletion layer and the equation of motion is integrated numerically. z_0, ϕ_S, and τ denote the width of the depletion layer, the surface potential, and the CBM lifetime, respectively. (a) z_0 is varied, (b) τ is varied, (c) ϕ_S is varied while keeping the other parameters constant.

differential equation is solved by numerical integration.

As can be seen from our data the bare pump pulse intensity $A_1(t)$ can be used as source term only for the case of the conduction band state CBS. For the conduction band minimum CBM, we have to take transport from the bulk into account: in this model the optical excitation by the pump pulse occurs over the whole penetration depth of the light, which is given by the inverse absorption coefficient of Cu_2O, $\alpha^{-1} = 37$ nm for the photon energy of 3 eV (the refractive index $n = 2.9$ and the extinction coefficient $k = 0.86$ at $\lambda = 400$ nm [8] translate into $\alpha = 4\pi k/\lambda = 2.7 \times 10^7$ m^{-1}). Due to light absorption electrons are promoted into all available conduction band states from where they decay very fast into the CBM. This leads to an effective source term $A_{\text{eff}}(z, t)$ for the CBM at $k = 0$ in momentum space and at depth z and time t. The amplitude of this source term is weighted with the pump light intensity at this depth:

$$A_{\text{eff}}(z,t) \;=\; A_{\text{eff}}(t)\, e^{-\alpha z}.$$

The electronic wavepacket is subject to the electric field generated by the surface depletion layer. The initial group velocity normal to the surface is assumed to be zero. For p-type Cu_2O, the surface band bending leads to acceleration of electrons $a(z,t)$ towards the surface:

$$a(z,t) \;=\; \frac{2\, e\, \phi_S}{m^\star\, z_0} \cdot \left(1 + \frac{z}{z_0}\right),$$

where $\phi(z) = \phi_S\,(1 - z/z_0)^2$ is the potential due to surface band bending, and $m^\star \approx 0.99\, m_e$ the effective band mass of the conduction band at Γ in units of the free-electron rest mass m_e [9]. Integrating the equations of motion allows the time to be computed which is required for the wavepacket to reach the surface. We emphasize that the experimental observations in 2PPE can only be explained assuming a mean-free path of the photoelectrons comparable to the one at VUV energies, thus of the order of 1 nm. This is negligible with respect to the width of the depletion layer which determines the depth out of which transport towards the surface is observed. Therefore, we consider the 2PPE signal to be proportional to the CBM population in a thin 1 nm thick layer at the surface.

The contribution of each layer is then weighted with the light intensity at this layer and the fraction of electrons which reaches the surfaces without inelastic scattering event, i.e. weighted with the factors

$$N_{\text{CBM}} \propto \; e^{-\alpha z} \; e^{-t/\tau},$$

where t is the time to reach the surface and τ the lifetime of the CBM, which can be obtained from experiment. All the contributions are summed up yielding the final transient. This transient is the effective source term for the conduction band population and is to be compared to the source term obtained by fitting the experimental transients.

In Fig. **S5** we show some transients N_{CBM} as function of delay time after the pump pulse for different parameter sets. The three parameters entering the calculation are the band bending parametrized by a width z_0 of the depletion layer and the surface potential ϕ_S, and the lifetime of electrons in the conduction band. We can observe that the transport produces a CBM population which is delayed and broadened. To a first approximation it yields an effective source term which can be modeled by a gaussian, shifted and broadened. Since the CBM lifetime is readily obtained from the experimental transients, the remaining two parameters z_0 and ϕ_S are the only free parameters. They can be related to known bulk properties like the density of acceptors and the dielectric constant [2] or compared to the surface core level shifts obtained from experiments as shown above.

Defect states

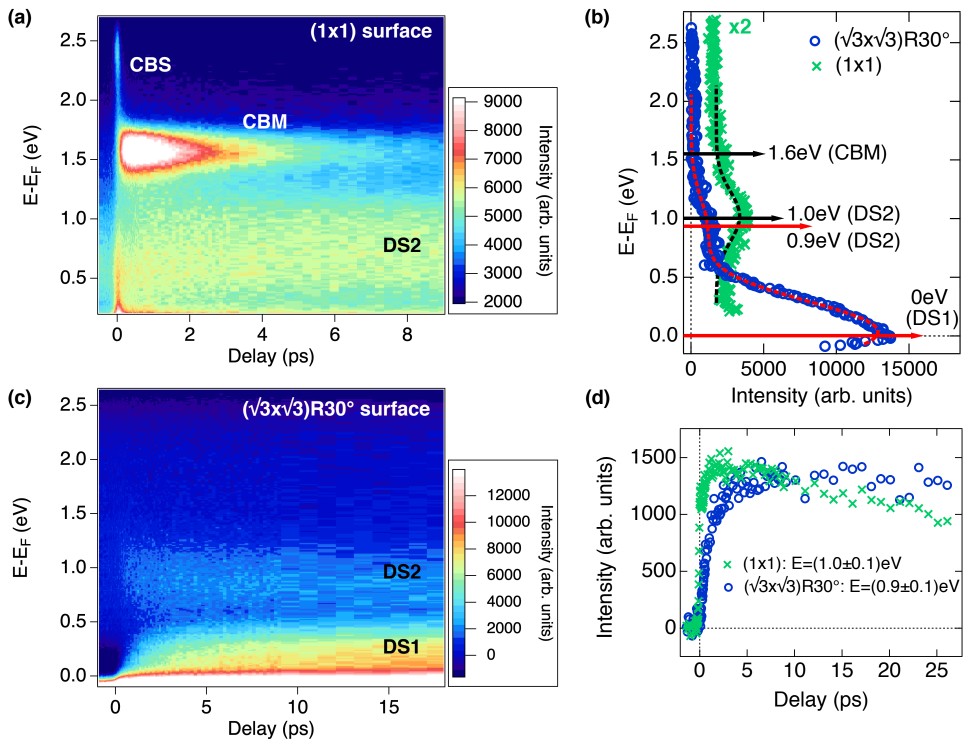

Supplementary Figure S6: **Preparation-dependent defect state.** (a) Time-dependent electron energy distribution for one typical preparation of $Cu_2O(111)$ with a (1×1) surface. The data were recorded with p-polarized 3 eV pump and 6 eV probe light. For comparison, an equivalent data set from a $\sqrt{3}$ surface is shown in (c). (b) Spectra taken 25 ps after excitation for both surfaces. Data from (1×1) surfaces are plotted as green crosses, from the $\sqrt{3}$ surface as open blue circles. The energy positions of the defect state DS2 are determined using gaussian fits; the fit results are included as dashed lines. (d) Transient population of the state DS2.

While the photoemission spectra of the valence and conduction bands as well as the conduction band dynamics were very reproducible between many preparations, an additional defect state (DS2) appeared on both, (1×1) and $\sqrt{3}$ surfaces with varying intensity. Examples for both surfaces are shown in Fig. S6. In order to determine the energy of DS2 while avoiding any influence of the conduction band population, spectra taken at long delays of 25 ps were analyzed using gaussians. In case of the (1×1) surface the state DS2 is found at a slightly higher energy of $E - E_F = 1.0$ eV ($E - E_{VBM} = 1.27$ eV) in comparison to its energy $E - E_F = 0.9$ eV ($E - E_{VBM} = 1.31$ eV) on the $\sqrt{3}$ surface. The energy positions related to the VBM position coincide within the error bars.

The time-dependent integrated intensity of DS2 on the (1×1) surface shows a rise on the timescale of roughly 1 ps followed by a slow recovery. In contrast to that, on the $\sqrt{3}$ surface containing a high density of ordered surface defects the intensity raise of the same state is slower reaching saturation within 5 ps. Despite the different dynamics in both cases we tentatively assign both states to similar defect states, like e.g. interstitial oxygen, based on the energy positions and preparation-dependent appearance. For the bulk-like (1×1) surface, where the surface conduction bands can be populated, a decay into this defect might explain the faster rise time at short delays, whereas the different decay times could be traced back to different amount of relaxation channels in both cases. Note that during all the experiments, we did not observe any influence of these defects on the conduction band population, like in particular trapping of electrons.

X-ray photoelectron spectroscopy

Core level spectra of Cu 2p, O1s and C1s taken using a non-monochromatized Mg Kα x-ray source are shown in Fig. **S7** from the (1×1) surface. A gaussian fit assuming a linear background was applied to the data to obtain the peak positions and peak areas which are listed in the table. From the Cu 2p spectrum the oxidation state of copper can be derived as Cu^{+} according to the exact binding energy and the satellite features. By comparing the intensity ratio of copper and oxygen after normalization to the corresponding photoemission cross sections [10] (see table) the stoichiometry can be determined to be between 1.65 using the Cu2p$_{1/2}$ peak or 1.84 for Cu2p$_{3/2}$. The deviation from the nominal stoichiometry of 2:1 can be rationalized by the oxygen termination of the (111)-surface and possibly diffraction effects, which enhance the oxygen intensity in normal emission (see supporting information of Ref. [4] for a more detailed discussion). The carbon 1s signal was used to check the cleanliness of a sample: during all experiments, its amplitude was kept below a specific level which corresponds to a carbon contamination of less than 4% of a monolayer. The peak positions and the Cu:O-ratio do not change with preparation after the first approximately 20 cleaning cycles.

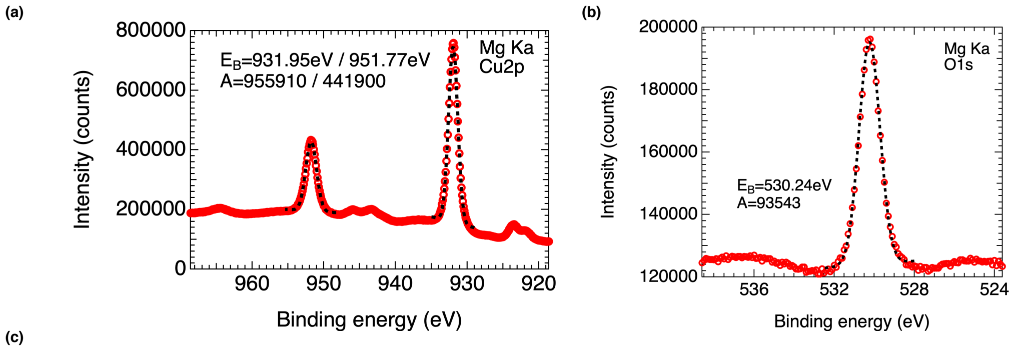

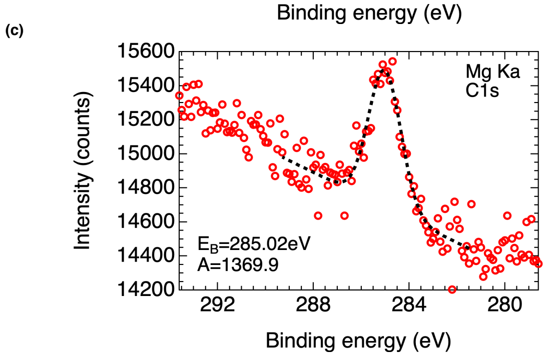

	E_B in eV	$I=A/\sigma$	I/I_{O1s}
**$Cu2p_{1/2}$**	951.77	54022.0	1.65
**$Cu2p_{3/2}$**	931.95	60233.8	1.84
**O1s**	530.24	32822.1	1
**C1s**	285.02	1369.9	0.04

Supplementary Figure S7: **XPS core level spectra.** Cu2p (a), O1s (b) and C1s (c) core level spectra of $Cu_2O(111)$ measured with a non-monochromatized Mg Kα x-ray source (1253.6 eV) from the (1×1) surface. The binding energies and peak areas obtained from a gaussian fit with linear background (black dashed line) are indicated in the graphs. In the table, we compile the binding energies and the integrated peak intensities after normalization to the related photoemission cross-sections [10].

References

[1] Smoluchowski, R. Anisotropy of the Electronic Work Function of Metals. *Physical Review* **60**, 661 (1941).

[2] Sze, S. *Semiconductor Devices: Physics and Technology* (John Wiley and Sons, New York, Chichester, Singapore, 1985).

[3] Omelchenko, S., Tolstova, Y., Atwater, H. & Lewis, N. Excitonic effects in emerging photovoltaic materials: A case study in Cu_2O. *ACS Energy Letters* **2**, 431–437 (2017).

[4] Leuenberger, D. *et al.* Atomically resolved band bending effects in a p-n heterojunction of Cu_2O and a cobalt macrocycle. *Nano Letters* **17**, 6620–6625 (2017). URL https://doi.org/10.1021/acs.nanolett.7b02486.

[5] Tanuma, S., Powell, C. J. & Penn, D. R. Calculation of electron inelastic mean free paths (IMFPs) VII. Reliability of the TPP − 2M IMFP predictive equation. *Surface and*

Interface Analysis **35**, 268–275 (2003). URL https://onlinelibrary.wiley.com/doi/abs/10.1002/sia.1526.

[6] Fröhlich, H., Pelzer, H. & Zienau, S. XX.Properties of slow electrons in polar materials. *The London, Edinburgh, and Dublin Philosophical Magazine and Journal of Science* **41**, 221–242 (1950). URL https://doi.org/10.1080/14786445008521794.

[7] Stolz, H., Schöne, F. & Semkat, D. Interaction of rydberg excitons in cuprous oxide with phonons and photons: optical linewidth and polariton effect. *New Journal of Physics* **20**, 023019 (2018). URL https://doi.org/10.1088%2F1367-2630%2Faaa396.

[8] Ioffe Institute, R. F., St. Petersburg. n, k database. http://www.ioffe.ru/SVA/NSM/nk/ (2019).

[9] Hodby, J. W., Jenkins, T. E., Schwab, C., Tamura, H. & Trivich, D. Cyclotron resonance of electrons and of holes in cuprous oxide, Cu_2O. *Journal of Physics C: Solid State Physics* **9**, 1429–1439 (1976). URL http://dx.doi.org/10.1088/0022-3719/9/8/014.

[10] Scofield, J. Hartree-slater subshell photoionization cross-sections at 1254 and 1487 eV. *Journal of Electron Spectroscopy and Related Phenomena* **8**, 129 – 137 (1976). URL http://www.sciencedirect.com/science/article/pii/0368204876800151.
